# Simultaneous Propionibacterium avidum and Propionibacterium acnes Chronic Periprosthetic Hip Joint Infection: A Case Report

**DOI:** 10.7759/cureus.20771

**Published:** 2021-12-28

**Authors:** Stefano Gussago, Luigia Elzi, Michele Arigoni, Cristiana Poroli Bastone, Mauro N Molina

**Affiliations:** 1 General Surgery, Ente Ospedaliero Cantonale, Ospedale Regionale di Locarno, Locarno, CHE; 2 Infectious Diseases, Ente Ospedaliero Cantonale, Ospedale Regionale di Bellinzona e Valli, Bellinzona, CHE

**Keywords:** co-infection, peri-prostethic hip infection, peri-prosthetic joint infection, propionibacterium avidum, propionibacterium acnes

## Abstract

Prosthetic hip joint replacement is considered the operation of the 20th century because of its wide diffusion and good outcome. More than 1 million prostheses are implanted worldwide annually. Although hip arthroplasty is considered a safe procedure, different complications can occur in relation with surgery. Periprosthesic joint infection is the most feared for its morbidity for the patients, and for the economic costs it generates. Most surgical site infections after hip arthroplasty are related to frequent germs as *Staphylococci* or *Enterobacteriaceae*, while *Propionibacterium* infections are more rare and often challenging in diagnosis and therapy. We report a case of a 77-year-old diabetic overweight male patient who developed a periprosthetic hip infection due to *P. avidum* and *P. acnes* after a mini-invasive direct anterior approach. To our knowledge, this represents the first case of chronic periprosthetic hip joint co-infection.

## Introduction

Prosthetic hip joint replacement is considered the surgery of the 20th century because of its wide diffusion and good outcome. More than 1 million prostheses are implanted worldwide annually. Although hip arthroplasty is considered a safe procedure, different complications can occur in relation with the surgery; periprosthetic joint infection (PJI) is the most feared complication due to its morbidity for the patients and the economic costs it generates. Most surgical site infections after hip arthroplasty are related to common germs such as *Staphylococci* or *Enterobacteriaceae* [1], whereas *Propionibacterium* infections are more rare and often challenging in terms of diagnosis and treatment.

*Propionibacterium acnes* is a Gram-positive, rod-shaped, slow-growing bacterium. It is a common skin commensal, but it has also been shown to be involved in the pathogenesis of acne vulgaris [2]. Although regarded as a low-virulence bacterium, *P. acnes* may be involved in PJI, especially in shoulder implants [3]. *Propionibacterium avidum* is a Gram-positive, anaerobic-aerotolerant, rod-shaped bacterium belonging to the class of Actinobacteria of the order Propionibacteriales, and it is considered a skin commensal with a specific tropism for moist areas [4]. Although there is increasing interest in this germ because of its capacity to cause opportunistic infections, most case reports are related to soft tissue abscess or native joint arthritis. Further study on *P. avidum* showed the presence of an exopolysaccharide (EPS)-like structure on atomic force microscopy, as suggested by the finding of an EPS biosynthesis gene cluster in the genome of *P. avidum*, indicating a certain possibility of virulence. In 2016, Peter Wildeman et al. described the first two cases of prosthetic hip joint infection due to *P. avidum* [5]; both patients described were morbidly obese, and the surgical approach was conducted via an anterolateral curved skin incision (Watson-Jones approach). Two other case series in 2018 confirmed a major prevalence of hip prosthesis infection due to *P. avidum* in patients with a high body mass index (BMI) who underwent arthroplasty via an anterior approach [6,7]. We report a case of a 77-year-old diabetic, overweight (BMI 29.8 kg/m^2^) male patient who developed a periprosthetic hip infection due to *P. avidum* and *P. acnes* after a mini-invasive direct anterior approach (DAA).

## Case presentation

A 77-year-old man was admitted for an elective right hip arthroplasty due to symptomatic osteoarthritis with progressive hip pain and rigidity (preoperative range of motion 0-5-85° in hip flexion/extension). The patient had a medical history of coronary heart disease, type II diabetes mellitus treated with metformin, and peripheral artery disease previously treated by left femoral angioplasty. The BMI at admission showed a moderate degree of overweight (BMI 29.8 kg/m^2^).

Right total hip arthroplasty with a non-cemented implant (Zimmer: Allofit cup and Alloclassic stem) using a minimally invasive single-incision DAA was performed. The skin was previously disinfected with povidone-iodine solution (Betaseptic), and an antibiotic prophylaxis with Cefazolin 2 g was administered intravenously 30 min before the skin incision. Due to significant preoperative hip rigidity, the surgical exposure was difficult, resulting in a prolonged surgical time of 2 h and important blood loss of 1150 mL. The wound was closed with a continuous intra-dermic absorbable suture over a sub-fascial drainage, which was removed after 24 h. No antibiotic therapy was administered in the postoperative period. The clinical course was uneventful, and the patient was transferred to the rehabilitation clinic after five days. The wound did not show any signs of infection at discharge. Histological examination of the joint capsule revealed the presence of chronic inflammation with a synovial cyst. Twenty days after surgery, the patient returned to our emergency department with suspicion of a wound infection. Clinical evaluation revealed the presence of a small non-penetrating cranial wound opening (1 cm) with the presence of fibrin on the wound bed. There were no other clinical signs of infection. A blood sample test showed an increased level of C-reactive protein (CRP, 128 mg/L) with a normal white cell count. Soft tissue ultrasound showed a deep anechoic collection located peripherally to the femur (maximal diameter 9 cm) and a small fluid component in the subcutaneous tissue under the surgical scar (maximal diameter 2 cm). Due to the absence of clear clinical signs of an infection, no antibiotic therapy was initiated. The culture examination of the punctured collection showed no bacterial growth, and consequently no antibiotic regimen was administered. Therefore, the patient first returned to the rehabilitation clinic, and after a further uneventful follow-up, he was discharged home. The subsequent follow-up was normal with no local signs of infection.

**Figure 1:**
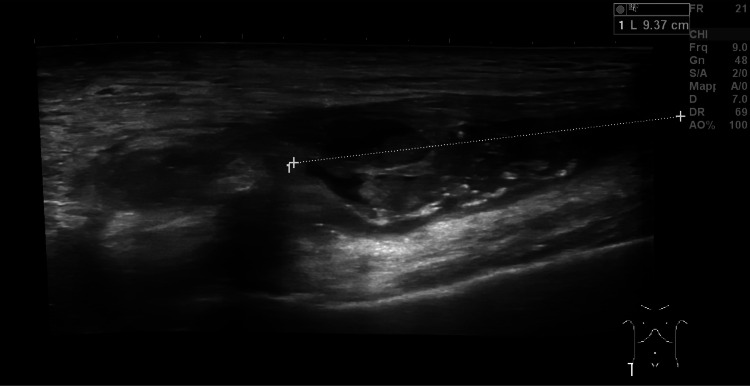
Preoperative ultrasound imaging of the periprosthetic fluid collection

**Figure 2:**
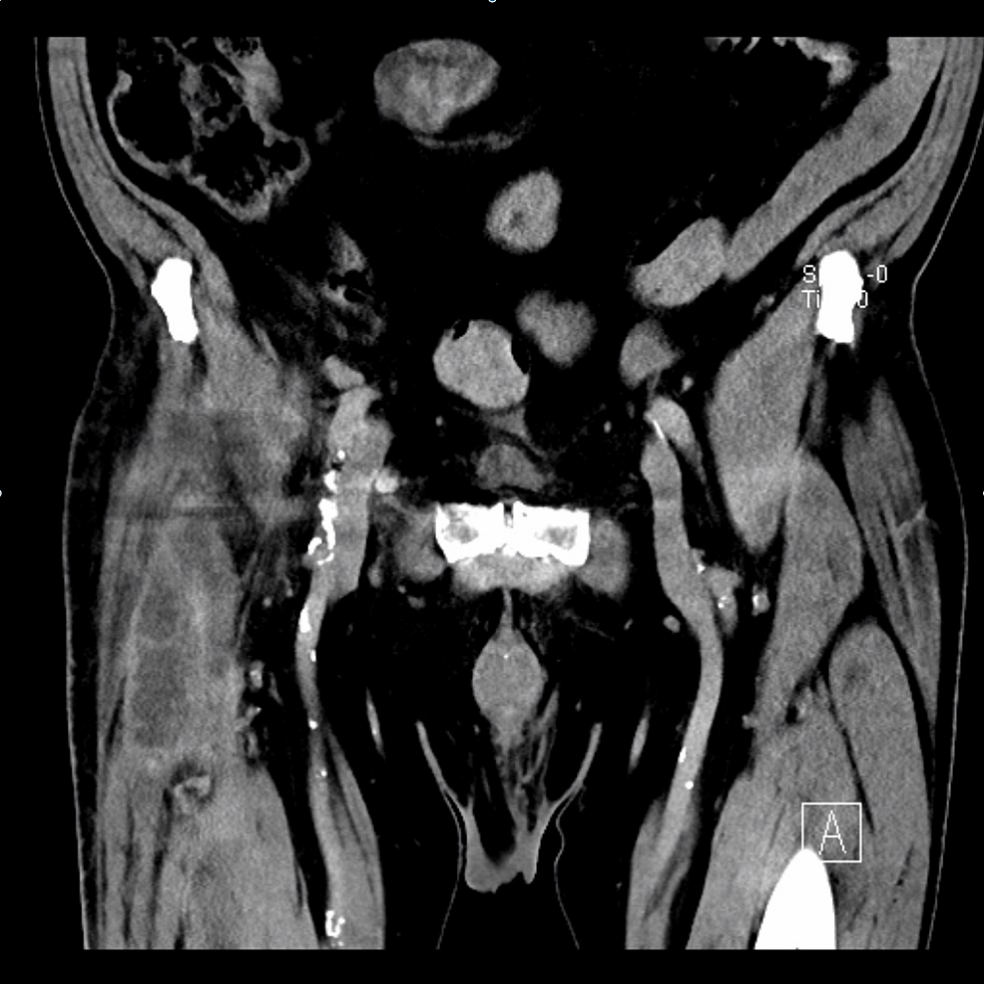
Preoperative contrast-enhanced CT scan of the periprosthetic fluid collection CT, computed tomography

Five months after surgery, the patient was referred again to our hospital due to profuse fatigue, discomfort, progressive weight loss (9 kg in the last three months), and night sweats. Blood tests showed a slightly elevated CRP level (12 mg/L) and moderate microcytic hypochromic anemia not related to iron deficiency. Erythrocyte sedimentation rate (ESR) was elevated at 84 mm/min. Clinical examination revealed mild hip pain during weight bearing and persistent swelling of the right groin. Ultrasound of the hip joint showed a collection with a maximal width of 10 cm and involvement of the joint, suspicious of a deep infection (Figure 1). Contrast-enhanced computed tomography (CT) of the hip confirmed the presence of the collection near the femur (Figure 2). In addition, the fluid was sampled by fine-needle aspiration, and culture examination revealed the growth of *P. avidum* sensitive to amoxicillin/clavulanic acid.

After interdisciplinary discussion, we proceeded to a two-stage surgical revision of the prosthesis. During the first surgery, conducted by the cranial extension of the previous surgical access (DAA), the collection was drained, and periarticular tissue was debrided and sampled for bacteriology; after removal of all prosthetic components (acetabular cup, femoral stem and head), an antibiotic-coated cement spacer (gentamycin-clindamycin) was inserted. Further bacteriological samples were obtained, and the removed material was sent for sonication. At the end of the bacteriological sampling, a single dose of daptomycin 500 mg IV was administered, and therapy with amoxicillin/clavulanic acid 2.2 g IV three times daily was started intraoperatively. After surgery, the patient received two red blood cell units for severe anemia. Bacteriological examination showed an extensive growth of *P. avidum*. This was confirmed in the sonicate of the prosthesis (104 colony-forming units [CFU]), which also revealed the presence of *P. acnes* (106 CFU) sensitive to amoxicillin/clavulanic acid (Table 1). 

**Table 1:** Patterns of the minimum inhibitory concentration values of Propionibacterium avidum and Propionibacterium acnes isolated

	Propionibacterium avidum	Propionibacterium acnes
Ceftriaxone	0.5 mg/L	0.25 mg/L
Levofloxacin	0.19 mg/L	0.19 mg/L
Rifampicin	0.004 mg/L	0.006 mg/L
Fusidic acid	2 mg/L	1 mg/L
Amoxicillin/clavulanic acid	0.25 mg/L	0.19 mg/L
Clindamycin	0.03 mg/L	Not tested

**Figure 3:**
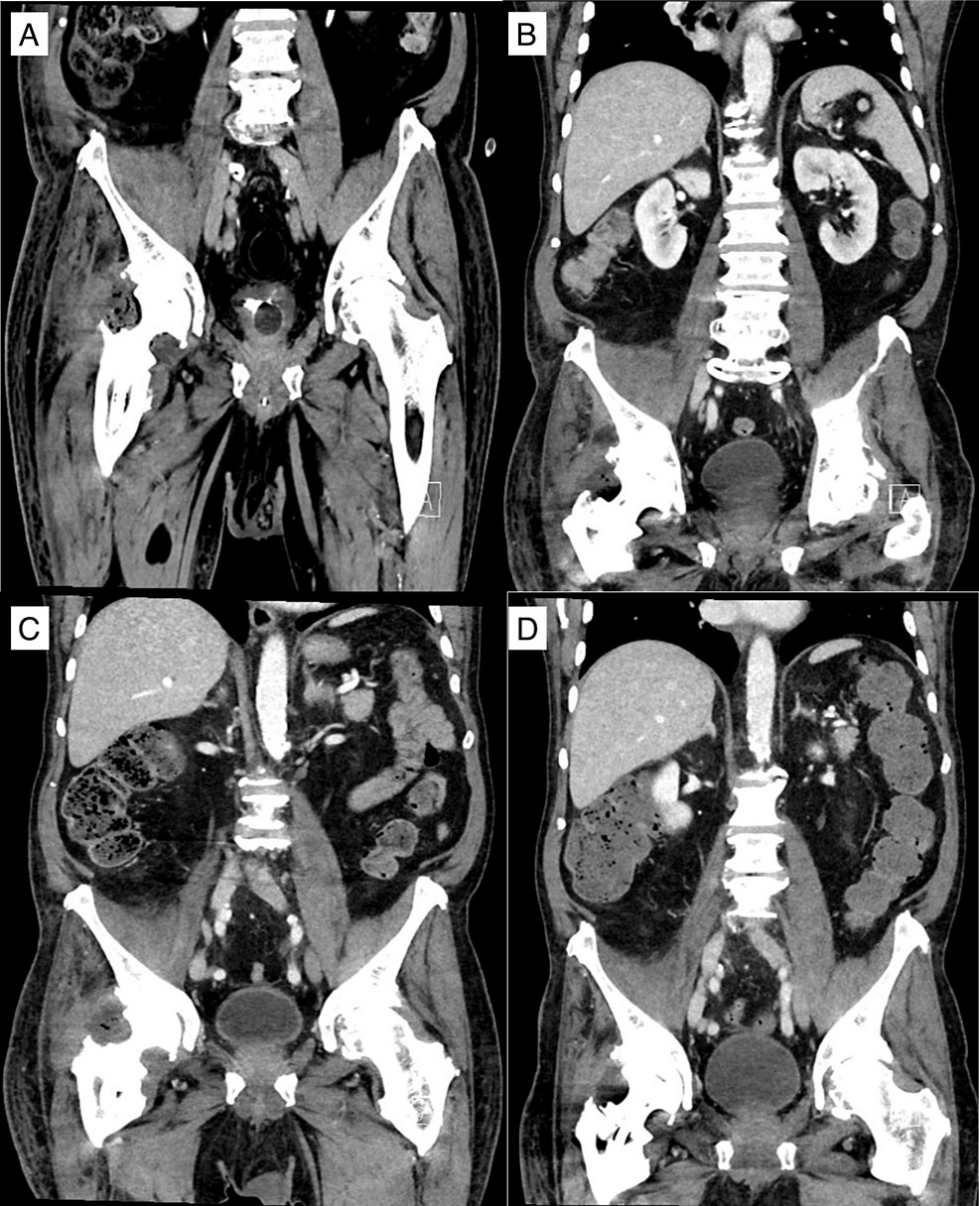
Radiological follow-up of the iliopsoas abscess at days 4 (A), 12 (B), 21 (C), and 56 (D) postoperatively

**Figure 4:**
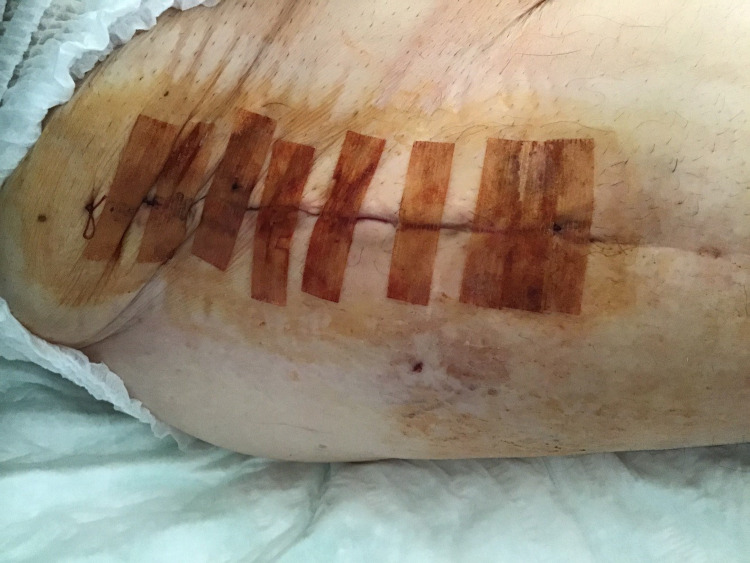
Surgical wound after the second stage of revision for periprosthetic joint infection

The antibiotic therapy with amoxicillin/clavulanic acid IV was administered for a total of four weeks in hospital before shifting to oral clindamycin 600 mg three times daily for an additional four weeks after discharge. A radiological follow-up by contrast-enhanced CT scan was performed to monitor the evolution of the right iliopsoas abscess, confirming a progressive reduction of the collection at days 4, 12, 21, and 56 after the first-stage surgery (Figure 3). At the time of discharge, the surgical wound appeared to be completely healed, and the patient could walk with crutches with partial weight bearing on the right hip. Eight weeks after the surgery to remove the infected prosthesis, the patient had a normal CRP level (5 mg/L) with no collection on the follow-up CT scan and was ready for the implant of the new prosthesis through the same surgical approach. An uneventful antibiotic holiday period of one week preceded the intervention. The removed spacer was sent for sonification, and different tissue biopsies were collected for bacteriological culture. After a single dose of daptomycin 700 mg IV, we implanted the new total hip prosthesis (De Puy: Pinnacle cup and Coral cemented stem) (Figure 4). The surgery was followed by antibiotic therapy with amoxicillin/clavulanic acid 2.2 g IV three times a day until negative results of the culture samples were obtained. The clinical course was characterized by persistent serous fluid secretion that was treated successfully with a superficial negative pressure treatment (Prevena^TM^). On the 14th postoperative day, the patient was finally transferred to the rehabilitation clinic. At the six-week and one-year follow-up, the patient showed a normal local status and no clinical or biological signs of recurrence of the infection.

## Discussion

The interest in understanding the biological behavior and pathological potential of *P. avidum* is progressively increasing in the last years [5]. The largest case series available in the literature suggest an increased incidence of *P. avidum* PJI in obese patients and after the use of an anterior surgical approach to the hip. No correlation was found with other classical PJI risk factors such as diabetes mellitus or immune suppression. A possible correlation to these findings is linked to the specific tropism of *P. avidum* for wet areas of the body, in this case the groin of pathologically obese patients. Although our patient had some classical risk factors for surgical site infection such as diabetes, he was only overweight and not obese, with a BMI of 29.8 kg/m^2^, far from the median of 34 kg/m^2^ found in the case series by Achermann et al. [6]. In our patient, the DAA used for the primary hip arthroplasty was the only real risk factor for developing *P. avidum* PJI. We used this approach for hip arthroplasty in more than 750 patients for the last three years, with a very low infection rate, and this case is our first and only case of *P. avidum* infection.

In line with the available literature, the presentation of the disease was not associated with septic arthritis, but rather with clinical (chronic local pain, profuse fatigue, weight loss, night sweats) and biological (elevated ESR, microcytic hypochromic anemia) signs of chronic inflammation [6,7].

Another peculiarity of our case is the simultaneous growth in the sonicated material of *P. acnes*, a skin commensal that is best known for its role in acne vulgaris. Although its virulence and association with PJI is well established, especially in shoulder implants [3], infection by this germ in hip arthroplasty is rare. As described in the literature, all the biological samples from the fine-needle evacuation of the femoral collection and the biopsies of the first revision were negative for *P. acnes*. Only the sonicated material, cultivated with an extended protocol (14 days) [8], was positive for this second microbiologic agent [9]. The low virulence of this bacterium makes it difficult to detect, and only sonication of the explanted material often leads to the diagnosis in previously misinterpreted aseptic implant loosening. Although polymicrobial PJI associated with *P. acnes* is known in the literature [10], to our knowledge, co-infection of *P. acnes* and *P. avidum* has only been previously reported once in a series of 15 cases [7].

A one-stage procedure for hip replacement in case of *P. avidum* PJI has been described in the literature, especially for acute infections [6]. In our case, the infection was diagnosed five months after hip arthroplasty and was thus to considered as chronic. For this reason, we preferred to use a two-stage procedure as recommended in the literature [6]. According to the standard of care [6,7], we used an eight-week period of antibiotic therapy (first with amoxicillin/clavulanic acid IV, then shifted to clindamycin per os) between the two surgeries.

## Conclusions

*P. avidum* periprosthetic hip infections are a rare entity in the current literature. In our case report, we describe the first case, to our knowledge, of chronic periprosthetic hip infection due to *P. avidum* and *P. acnes* after a mini-invasive DAA, successfully treated with a two-stage procedure. Considering the rarity of this condition, our work might help in the management of similar cases.
